# Intensive combined balneotherapy and aquatic exercise for knee osteoarthritis: short-term clinical and functional outcomes

**DOI:** 10.3389/fmed.2026.1790566

**Published:** 2026-02-16

**Authors:** Gianluca Regazzo, Paola Contessa, Beatrice Forcato, Maddalena Fornasiero, Matteo Gaiofatto, Manuela Gibellini, Federico Massimo, Erika Venturini, Anna Scanu, Stefano Masiero

**Affiliations:** 1 Section of Rehabilitation, Department of Neuroscience-DNS, University of Padova, Padova, Italy; 2 Department of Neuroscience, Physical Medicine and Rehabilitation School, University of Padua, Padova, Italy

**Keywords:** balneotherapy, health resort, hydrokinesitherapy, knee OA, knee osteoarthritis, pain, quality of life, treatment efficacy

## Abstract

**Background:**

Knee osteoarthritis (OA) is a major cause of global disability, necessitating cost-effective interventions. While balneotherapy and acquatic exercise (AE) are established conservative treatments, evidence regarding their combined efficacy remains limited. This study evaluated the clinical impact of a combined program that include balneotherapy and AE in natural mineral waters - compared to balneotherapy alone in patients with mild-to-moderate knee OA in Health Resorts. Primary outcomes included pain intensity, joint range of motion (AROM/PROM), and functional indices [Western Ontario and McMaster Universities (WOMAC), Lequesne’s Algofunctional Index for Knee (LAI-knee)]. Secondary outcomes encompassed quality of life (Short Form-12) and psychological well-being (Pittsburgh Sleep Quality Index and Psychological General Well-Being Index).

**Methods:**

66 patients were allocated to either an experimental group (EG), receiving a combined two-week protocol of balneotherapy and AE in salt-bromine-iodine thermal water, or a control group (CG), receiving balneotherapy alone.

**Results:**

Both groups demonstrated significant short-term improvements in all the assessments included in study. However, the EG exhibited a superior reduction in pain intensity (31% vs. 13% in CG) and more consistent gains in bilateral active range of motion (AROM). Linear mixed-effects models confirmed significant time effects for WOMAC, LAI, and SF-12 Physical Component scores for both groups. Regression analysis revealed that higher BMI and age negatively correlated with mobility gains.

**Conclusion:**

A combined intervention significantly enhances the analgesic and functional benefits of standard balneotherapy. By leveraging physical properties of mineral water, the combined protocol addresses both the mechanical and biological components of knee OA. These findings support the integration of water exercise with balneotherapy in Health Resorts for degenerative joint diseases and the personalization of treatment based on patients age and BMI.

## 1 Introduction

Knee osteoarthritis (OA) is a chronic degenerative joint disease characterized by the progressive loss of articular cartilage and concomitant subchondral bone remodeling (1). It represents a leading global cause of chronic pain and disability, imposing a substantial socioeconomic burden on healthcare systems (2). The prevalence of the condition is rising significantly, particularly among the elderly population: radiographic evidence of knee OA is present in 3.8% of the global population and estimates indicate that the number of individuals with symptomatic knee OA will increase by 50% by 2040 (3, 4).

While traditionally classified as a “wear-and-tear” non-inflammatory arthritis, current evidence highlights the role of low-grade chronic synovial inflammation (5). This process is driven by the intra-articular release of pro-inflammatory cytokines, matrix metalloproteinases (MMPs), and other catabolic mediators (6). Patients typically present with activity-related joint pain, morning stiffness, reduced range of motion (ROM), and joint effusion, which collectively impair physical function and diminish quality of life.

Among conservative treatment strategies, physical therapy, weight management, drug therapy, and patient education are considered the cornerstone of early-stage knee OA management (7–9). However, given the increasing prevalence of this chronic joint disorder and its associated disabilities, as well as the considerable social cost and pressure on health care structures, there is a pressing need to explore new treatment options and settings (10). In this context, Health Resort Medicine - including balneotherapy and acquatic exercise (AE) - has gained prominence for managing degenerative musculoskeletal conditions due to its favorable safety profile and therapeutic efficacy (11, 12).

Balneotherapy (or Crenotherapy) i.e., the use of (natural) mineral waters, gases and peliods constitutes a widely utilized in therapy of musculoskeletal disorders, primarily involving total or partial body immersion. The actions are linked to the specific chemical constitution and temperature at the source. The thermal effect plays a pivotal role in the immediate relief of symptoms; by inducing peripheral vasodilation, it enhances local blood flow, facilitates the washout of algogenic substances, and promotes significant muscle relaxation through the modulation of gamma-motor neuron activity (13). Beyond the thermal effect, the chemical–physical properties of mineral waters can modulate inflammatory markers and antioxidant pathways, leading to symptom relief, improved joint function and enhanced quality of life (11). Systematic reviews have demonstrated significant improvements in pain scores and quality of life in patients with OA of the knee, hip, and spine (14). Despite these promising data, thermal interventions are currently only “conditionally recommended” by international guidelines due to the heterogeneity of study designs and the scarcity of high-quality randomized controlled trials (RCTs) (15, 16).

AE, i.e., the use of water-based exercise in (natural) mineral waters, utilizes the physical properties of water, such as buoyancy, which reduces joint loading by decreasing apparent body weight, and hydrodynamic friction, which provides calibrated resistance for muscle strengthening, to obtain therapeutic effects (17). AE has proven beneficial for improving ROM and strength in various neurological and musculoskeletal disorders, and several studies have confirmed that water-based exercise yields comparable effects to land-based interventions for pain relief and functional improvement (18–22). A recent scoping review reported that AE is an effective intervention for managing knee OA, offering short-term significant improvements in key clinical outcomes (23).

Interestingly, evidence regarding the additional effects of combining AE with balneotherapy in muscle skeletal disorders remains limited. Preliminary data from our group on obese patients with knee OA suggested that AE in salt-bromine-iodine thermal water could provide sustained functional benefits for up to six months (24). However, to the best of our knowledge, a direct comparison between balneotherapy alone and a combined approach is still lacking. Therefore, the objective of the present study is to evaluate and compare the efficacy of standard balneotherapy versus a combined protocol of water-based exercise in salt-bromine-iodine thermal water and balneotherapy in patients with mild-to-moderate knee OA. Primary outcomes include pain intensity, joint range of motion and algo-functional indices. Secondary outcomes encompasses quality of life and psychological well-being evaluation.

## 2 Materials and methods

This randomized controlled trial was conducted at the Neurorehabilitation Unit of the University Hospital of Padua, Italy, following the principles of the Declaration of Helsinki. The protocol was approved by the Ethics Committee of the University-General Hospital of Padova (protocol number AOP2925, CET-ACEV: 5879/AO/23, study ID: 22564). All participants provided written informed consent prior to inclusion in the study.

### 2.1 Participants

**Figure 1:**
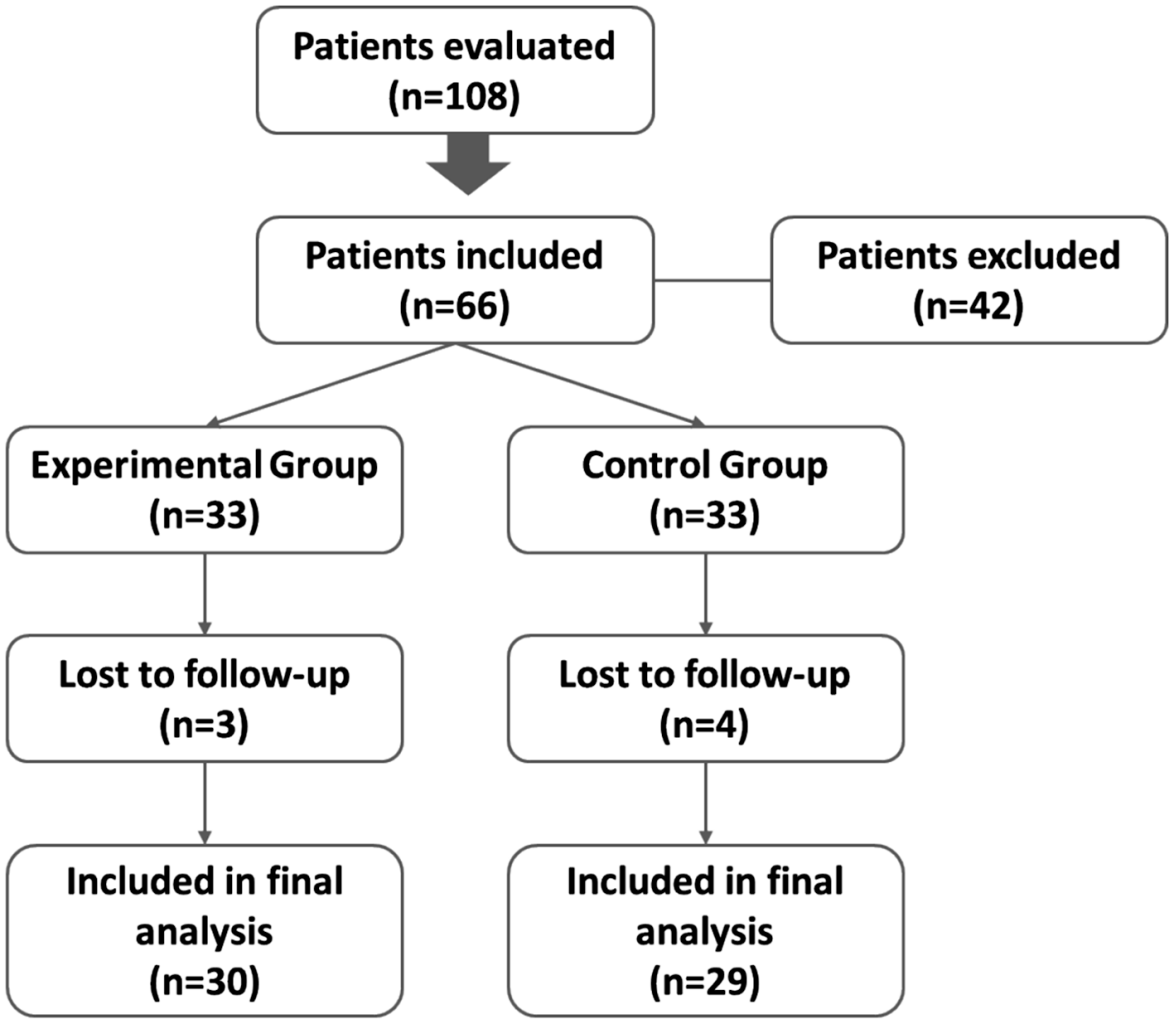
Flow diagram of the patient selection process.

A total of 108 male and female patients with knee OA, screened at the Neurorehabilitation Unit of the University-General Hospital of Padova (Italy), were assessed for eligibility. Inclusion criteria comprised individuals aged 18–80 years with a confirmed diagnosis of grade II–III knee OA according to the Kellgren-Lawrence (K-L) scale. Additional inclusion criteria were the absence of Health Resort treatment within the last 12 months and of rehabilitative or infiltrative treatment within the last 6 months. Patients who could not understand and sign informed consent or who had a body temperature exceeding 37.5° C, with a history of epilepsy, severe psychiatric disease, diagnosed neoplasm, or pregnancy; and patients with skin infections, open wounds, systemic inflammation, significant organ dysfunction (such as heart, liver, respiratory, or kidney failure), and those with urinary or fecal incontinence (contraindication to health resort treatment) were also excluded. Of the 108 screened participants, 42 were excluded for failing to meet the inclusion criteria, while the remaining 66 individuals were successfully randomized into the experimental group (EG; n = 33) or the control group (CG; *n* = 33) on a 1:1 basis. Throughout the duration of the study, three subjects from the experimental group and four from the control group failed to complete the follow-up assessments and were consequently classified as dropouts (Figure 1).

### 2.2 Intervention

The EG underwent a combined rehabilitation protocol consisting of balneotherapy and AE, while the CG received balneotherapy as a stand-alone intervention. Treatments were administered over a two-week period in Health Resort facilities located within the Euganean Basin in Veneto Region, Italy. All facilities were accredited by the National Health Service, and patients underwent treatment on an outpatient basis. To ensure the highest level of standardization across the different facilities and to minimize inter-operator variability, all clinicians and physiotherapists involved in the study attended dedicated training sessions to align on the specific delivery of the intervention protocols.

The balneotherapy protocol, which was identical for both groups, comprised 12 sessions distributed over two consecutive weeks. Each individual session involved the application of mature therapeutic mud (peloid) for 20–25 min (42°–45 °C), followed by a 15–20 min thermal bath. The immersion took place in sodium chloride–bromide–iodide thermal water maintained at a temperature of approximately 36°–38 °C (Table 1).

**Table 1:** Characteristics of sodium chloride–bromide–iodide thermal water.

Characteristic	Description/value
Chemical type	Sodium chloride–bromide–iodide Hyperthermal Water (rich in salt, bromine, and iodine).
Thermal class	Hyperthermal (Natural temperature > 40 °C).
pH	7.1 (Neutral/Slightly Alkaline).
Fixed residue (180 °C)	5–6 g/L (Rich in dissolved minerals).
Sodium (Na^+^)	~ 1.2 g/L, main cation
Chloride (Cl^−^)	~ 2.1 g/L, main anion
Bromine (Br^−^)	~14 mg/L
Iodine (I^−^)	~0.8 mg/L
Others	Calcium, Magnesium, Potassium, Silica, Sulfates, Bicarbonates.

In addition to balneotherapy, participants in the experimental group attended supervised AE sessions for a total of 12 sessions over two weeks. These sessions lasted 40 min each and were conducted in groups of four patients to ensure safety and personalized supervision by a physical therapist. The AE program was specifically structured to target the functional deficits associated with knee OA, focusing on the strengthening of the lower limb musculature, the restoration of joint range of motion, and the improvement of overall coordination and balance. The detailed program is further described in Table 2.

**Table 2:** Aquatic exercise program.

Exercise	Duration	Detailed description
Water walking	15 min	Forward, backward, and sideways movements to improve joint mobility and coordination
Knee lifts	5 min	Alternating knee rises to a 90° angle while standing to maintain balance
Knee extensions	5 min	Seated leg extensions to strengthen the quadriceps
Lateral lunges	5 min	Lateral leg rises while standing to strengthen abductor muscles
Stretching	5 min	Quadriceps and calf stretch to maintain muscle elasticity
Water bicycle	5 min	Simulated pedaling to enhance muscle mobility and endurance

### 2.3 Assessment

Clinical and functional evaluations were conducted at two distinct time points: a baseline assessment prior to the intervention (T0) and a follow-up evaluation performed one month after the completion of the treatment protocol (T1). These assessments encompassed a comprehensive battery of clinical, functional, and psychometric parameters designed to capture the multi-dimensional impact of the intervention on the participants’ health status. At T0 anthropometric and demographic data were collected for all participants, including age, gender, height, weight, body mass index (BMI), and the onset of symptoms. Other assessments were performed at both T0 and T1:

- Hand Grip Strength: measured using a digital handheld dynamometer for both dominant and non-dominant hands to assess systemic physical status.
- Knee goniometry: objective recording of the Active Range of Motion (AROM) and Passive Range of Motion (PROM) of the knee.
- Numerical Rating Scale (NRS): a 0–10 scale quantifying perceived pain intensity (0 = no pain, 10 = worst possible pain).
- Western Ontario and McMaster Universities Osteoarthritis Index (WOMAC Index): a self-administered tool assessing symptoms over the previous 48 h across three subscales: pain (5 items), stiffness (2 items), physical functioning (17 items, including activities like walking and stair climbing).
- Lequesne Algo-functional Index (LAI): an aggregate measure of severity based on pain, maximum walking distance, and limitations in daily living activities.
- SF-12 Health Survey: monitors health-related quality of life via two main summaries, PCS (Physical Component Summary) and MCS (Mental Component Summary).
- Pittsburgh Sleep Quality Index (PSQI): self-reported measure of sleep quality (scores ranging from 0 to 21; scores >5 indicate significant sleep disturbances).
- Psychological General Well-Being Index (PGWBI): a 22-item assessment of psychological health, including anxiety, depressed mood, vitality, and self-control.

### 2.4 Statistical analysis

Variables were assessed at baseline (T0) and post-treatment (T1). Normality of data distribution was tested using the Shapiro-Wilcoxon test. Depending on the distribution, either parametric or non-parametric tests were employed for the analyses. Specifically, group differences in baseline (i.e., at T0) anthropometric, functional, and clinical outcome measures were assessed by a t-test in the case of normally distributed data, by Wilcoxon–Mann–Whitney test in the case of non-normally distributed data, or by Chi-squared tests in the case of categorical data. Group differences before (i.e., at T0) vs. after (i.e., at T1) intervention in functional and patient-reported clinical outcome measures were assessed by a t-test in the case of normally distributed data or by Wilcoxon-signed rank in the case of non-normally distributed data. Effect sizes are reported as Cohen’s d for parametric tests and rank-biserial correlation (r) using bias-corrected bootstrap resampling for non-parametric tests. For categorical variables (e.g., sex), effect size was quantified using Cramér’s V. Effect sizes were interpreted as small, medium, and large using conventional thresholds: Cohen’s d = 0.20, 0.50, and 0.80; rank-biserial correlation r = 0.10, 0.30, and 0.50; and Cramér’s V = 0.10, 0.30, and 0.50. Ninety-five percent confidence intervals were calculated for all effect size estimates to assess the precision of the observed differences. Continuous variables are reported as mean (standard deviation) or as median (interquartile range) depending on the distribution, and categorical variables as counts and percentages.

Linear mixed effects regression models were used to examine changes in outcomes between time points (i.e., T0 vs. T1) while accounting for interindividual variability and participant characteristics. Functional and patient-reported outcome measures of function, quality of life, and psychological parameters were entered as dependent variables. Fixed effects included time, Group, time*Group interaction, as well as participants’ age and BMI. Random intercepts for each participant were modeled to account for interindividual differences in baseline outcome levels. Age and BMI were standardized across participants. Groups were coded as EG = 1 (individuals who participated in the combined AE and balneotherapy intervention) and CG = 0 (individuals who participated in the balneotherapy intervention only).

Therefore, the main effect of time represents the change from time T0 to T1 for participants in the CG: a significant *p*-value indicates a significant change in the variable over times within CG participants. The main effect of Group represents the difference between the two groups (EG vs. CG) at baseline (T0): a significant *p*-value indicates a significant baseline difference between groups. The time*Group interaction tests whether the effect of Time differs between Groups (for EG relative to CG): a significant *p*-value indicates that the change from T0 to T1 differs between EG and CG participants, and it effectively indicates the improvement of EG relative to CG. The effect of Age represents the association between Age and the outcome variable: a significant *p*-value indicates that age is significantly associated with the outcome variable. Similarly, the term BMI represents the association between BMI and the outcome variable: a significant p-value indicates that BMI is significantly associated with the outcome variable.

## 3 Results

### 3.1 Baseline characteristics

**Figure 2:**
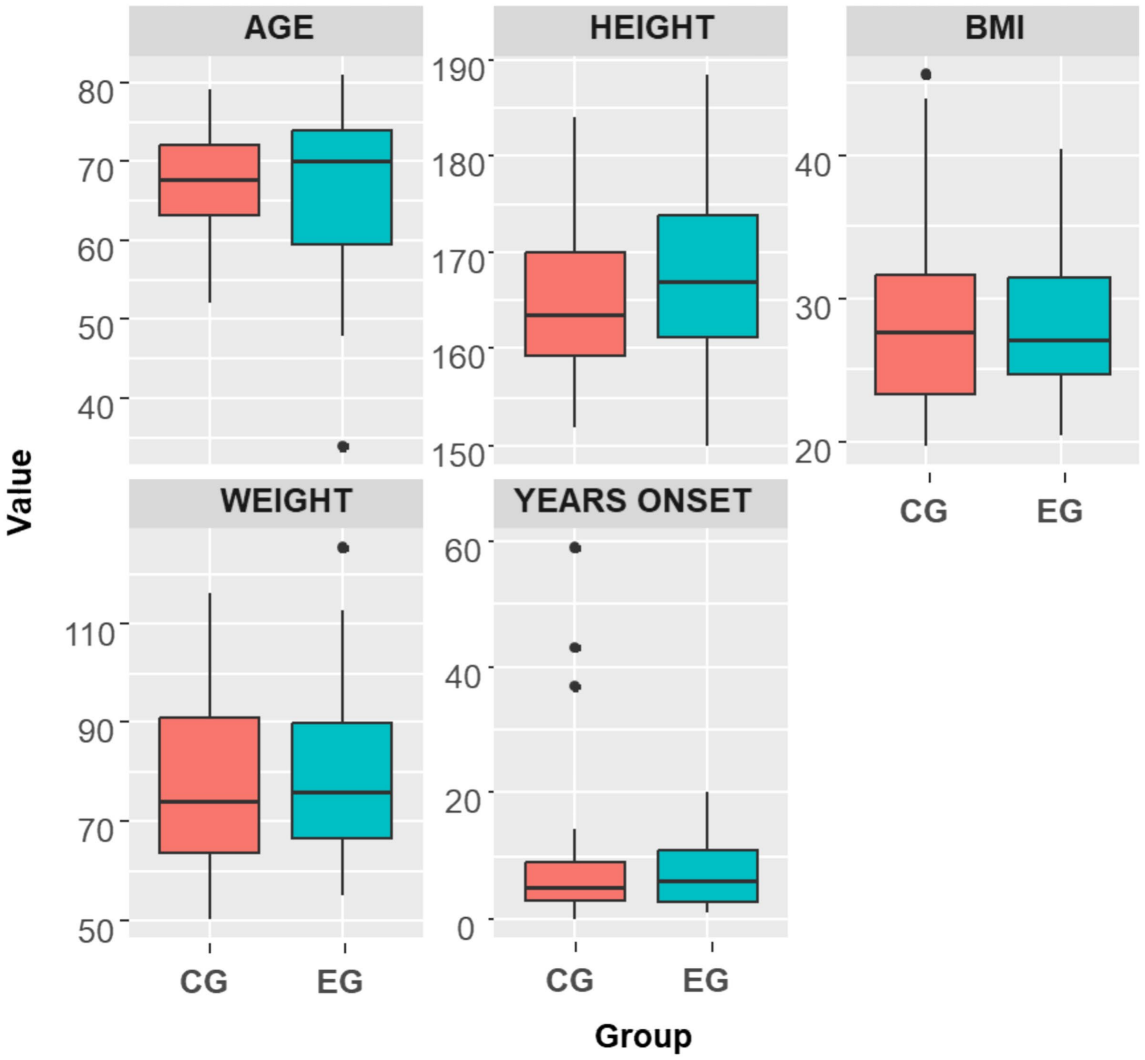
Baseline comparison (T0) in demographic measures between control group (CG) and experimental group (EG).

**Figure 3:**
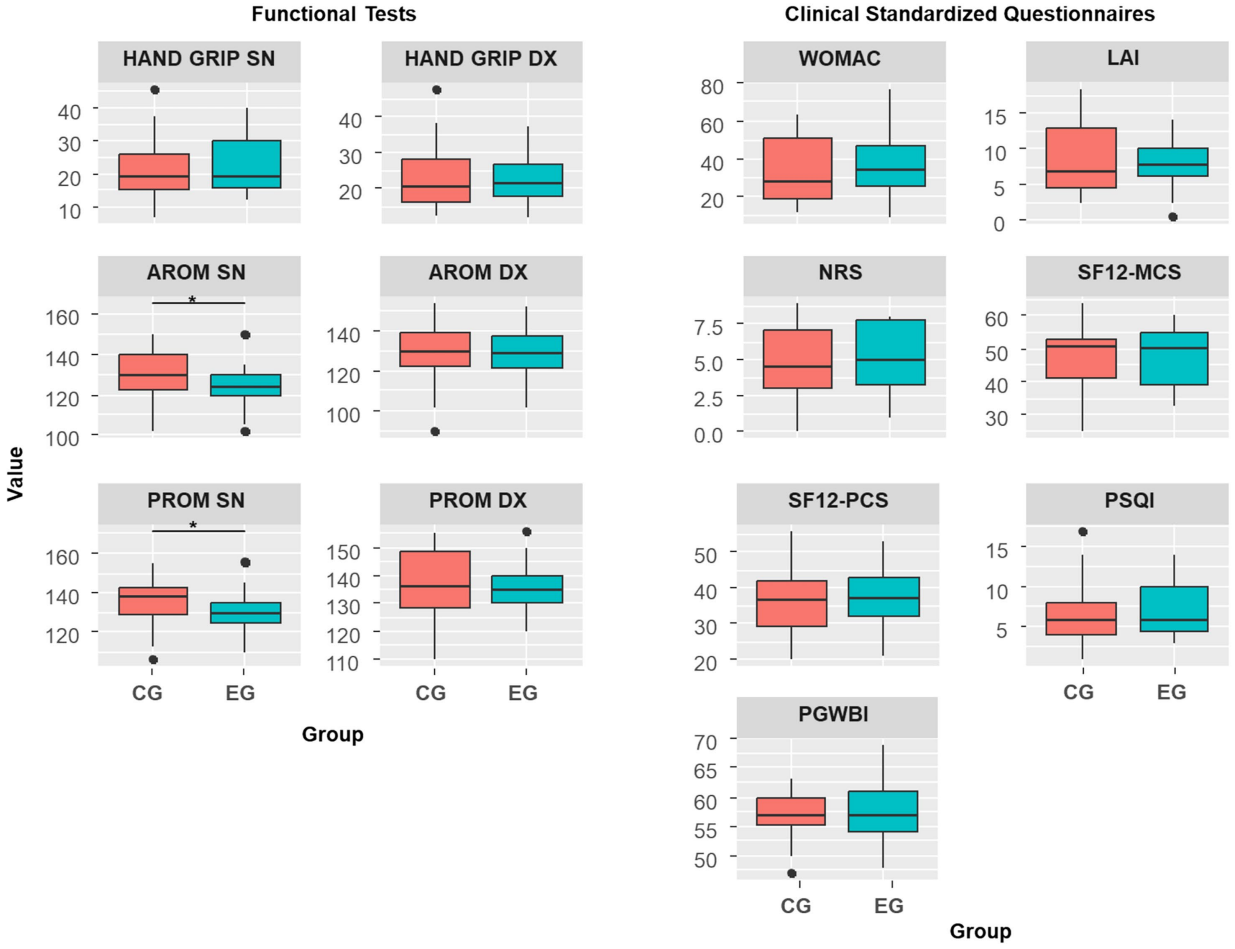
Baseline comparison (T0) in functional and clinical outcome measures between control group (CG) and experimental group (EG). Statistically significant differences between groups are indicated with an asterisk (*). AROM: active range of motion. PROM: passive range of motion. NRS: Numerical Rating Scale. WOMAC: Western Ontario and McMaster Universities Osteoarthritis Index. LAI: Lequesne Algo-functional Index. SF12-PCS: Physical component summary of the SF-12 Health Survey. SF12-MCS: Mental component summary of the SF-12 Health Survey. PSQI: Pittsburgh Sleep Quality Index PGWBI.

At the baseline evaluation, no statistically significant differences, with small effects sizes, were observed between the two groups regarding anthropometric measures or patient-reported clinical outcome measures (Table 3). However, a specific baseline discrepancy was identified for the left knee’s range of motion, where the EG exhibited significantly lower values compared to the CG for both active range of motion (123° ± 10° vs. 130° ± 13°; *p* = 0.010) and passive range of motion (130° ± 10° vs. 136° ± 11°; p = 0.033) (Table 4; Figures 2, 3).

**Table 3:** Baseline measures of control group (CG) and experimental group (EG).

Variable	Control group	Experimental group	*p*-value	Effect size (95% CI)
Sex	21 females (72%) 8 males (28%)	19 females (63%) 11 males (37%)	0.640	V = 0.1
Age (years)	68 (9)	66 (14)	0.885	rrb = 0.02 [0–0.05]
Height (cm)	164.8 (8.5)	167.8 (9.0)	0.204	d = −0.33 [−0.91–0.15]
Weight (kg)	76.76 (17.29)	80.73 (19.31)	0.408	d = −0.22 [−0.73–0.35]
BMI	27.5 (8.6)	27.0 (6.7)	0.974	rrb = 0.06 [0–0.19]
Years from Onset	5 (7)	6 (9)	0.621	rrb = 0.10 [0.001–0.30]

Continuous variables are reported as mean (standard deviation) or as median (interquartile range) depending on the distribution, and categorical variables as counts and percentages. Group differences are assessed by a t-test in the case of normally distributed data, by Wilcoxon–Mann–Whitney test in the case of non-normally distributed data, or by Chi-squared tests in the case of categorical data. Effect sizes are reported as Cohen’s d for parametric tests and rank-biserial correlation (rrb) for non-parametric tests. For categorical variables (e.g., sex), effect size is quantified using Cramér’s V. Ninety-five percent confidence intervals are calculated for all effect size estimates to assess the precision of the observed differences.

**Table 4:** Differences in values of all functional and patient-reported clinical outcome measures between T0 and T1 for both control group (CG) (*N* = 29) and experimental group (EC) (*N* = 30).

Variable	Group	T0	T1	*p*-value	Effect size (95% CI)
Hand Grip Dx (kg)	CG	21.5 (12.0)	19.9 (8.5)	0.134	rrb = 0.29 [0.07–0.67]
	EG	21.6 (9.1)	23.0 (11.1)	0.604	rrb = 0.10 [0.0001–0.31]
	*p*-value	0.891	0.554		
	Effect size (95% CI)	r = 0.02 [0–0.07]	r = 0.08 [0.001–0.25]		
Hand Grip Sn (kg)	CG	19.5 (12.2)	18.8 (7.5)	0.296	rrb = 0.20 [0.10–0.70]
	EG	19.1 (14.7)	20.6 (14.9)	0.737	rrb = 0.06 [0–0.20]
	*p*-value	0.705	0.628		
	Effect size (95% CI)	r = 0.05 [0–0.17]	r = 0.06 [0–0.21]		
AROM Dx (°)	CG	130 (16)	136 (11)	0.013*	d = −0.49 [−0.80 – −0.17]
	EG	129 (11)	134 (8)	0.031*	d = −0.42 [−0.72 – −0.09]
	*p*-value	0.785	0.376		
	Effect size (95% CI)	d = 0.07 [−0.46–0.61]	d = 0.23 [−0.32–0.78]		
AROM Sn (°)	CG	130 (16)	132 (10)	0.211	rrb = 0.20 [0–0.05]
	EG	124 (10)	130 (13)	0.005**	rrb = 0.50 [0.27–0.69]
	*p*-value	0.010*	0.245		
	Effect size (95% CI)	r = 0.34 [0.10–0.56]	r = 0.15 [0.007–0.39]		
PROM Dx (°)	CG	137 (12)	142 (10)	0.002**	d = −0.63 [−0.95–0.33]
	EG	136 (8)	140 (8)	0.031*	d = −0.42 [−0.78 – −0.08]
	*p*-value	0.724	0.386		
	Effect size (95% CI)	d = 0.09 [−0.45–0.65]	d = 0.23 [−0.32–0.76]		
PROM Sn (°)	CG	136 (11)	142 (9)	0.001**	d = −0.66 [−1.03 – −0.37]
	EG	130 (10)	135 (10)	0.011*	d = −0.50 [−0.98 – −0.12]
	*p*-value	0.033*	0.006**		
	Effect size (95% CI)	d = 0.58 [0.02–1.27]	d = 0.74 [0.24–1.27]		
NRS	CG	4.0 (3.5)	3.0 (2.0)	0.039*	rrb = 0.42
	EG	5.0 (4.5)	3.0 (3.0)	0.001***	rrb = 0.64
	*p*-value	0.349	0.661		
	Effect size (95% CI)	r = 0.13 [0.004–0.36]	r = 0.06 [0–0.20]		
WOMAC	CG	28.0 (29.0)	22.0 (16.0)	<0.001***	rrb = 0.75 [0.69–0.89]
	EG	34.0 (21.3)	22.5 (21.5)	<0.001***	rrb = 0.69 [0.53–0.84]
	*p*-value	0.275	0.982		
	Effect size (95% CI)	r = 0.14 [0.008–0.39]	r = 0.004 [0–0.002]		
LAI	CG	8.0 (4.6)	5.4 (3.3)	0.009**	d = 0.65 [0.30–1.08]
	EG	7.0 (3.2)	5.0 (3.1)	0.017*	d = 0.70 [0.21–1.74]
	*p*-value	0.779	0.479		
	Effect size (95% CI)	d = 0.08 [−0.57–0.68]	d = 0.22 [−0.41–0.86]		
SF12-MCS	CG	47.0 (21.0)	46.5 (12.3)	0.581	rrb = 0.07 [0.01–0.49]
	EG	47.6 (16.2)	54.5 (16.1)	0.063	rrb = 0.34 [0.30–0.66]
	*p*-value	0.806	0.043*		
	Effect size (95% CI)	r = 0.03 [0–0.12]	r = 0.27 [0.03–0.50]		
SF12-PCS	CG	37.5 (9.3)	41.2 (7.5)	0.041*	d = −0.43 [−0.83 – −0.04]
	EG	37.7 (8.1)	39.9 (7.6)	0.014*	d = −0.32 [−1.09–0.04]
	*p*-value	0.928	0.186		
	Effect size (95% CI)	d = −0.02 [−0.56–0.54]	d = 0.35 [−0.12–0.91]		
PSQI	CG	6.0 (4.3)	6.0 (3.0)	0.287	rrb = 0.21
	EG	6.0 (5.5)	6.0 (5.0)	0.231	rrb = 0.15
	*p*-value	0.458	0.603		
	Effect size (95% CI)	r = 0.11 [0.002–0.33]	r = 0.07 [0.001–0.24]		
PGWBI	CG	56.8 (3.9)	58.2 (4.4)	0.066	d = −0.36 [−0.79–0.02]
	EG	57.2 (5.0)	58.1 (3.2)	0.310	d = −0.19 [−0.58–0.18]
	*p-*value	0.708	0.914		
	Effect size (95% CI)	d = −0.10 [−0.61–0.44]	d = 0.03 [−0.52–0.60]		

Data are presented as mean (standard deviation) or as median (interquartile range) depending on the distribution. Group differences are assessed by a t-test in the case of normally distributed data, or by Wilcoxon–Mann–Whitney test in the case of non-normally distributed data. Effect sizes are reported as Cohen’s d for parametric tests and rank-biserial correlation (r) for non-parametric tests. Ninety-five percent confidence intervals are calculated for all effect size estimates to assess the precision of the observed differences. Asterisks indicate the level of statistical significance: *indicates *p* < 0.05, **indicates *p* < 0.01, ***indicates *p* < 0.001.

### 3.2 Within-group changes

**Figure 4:**
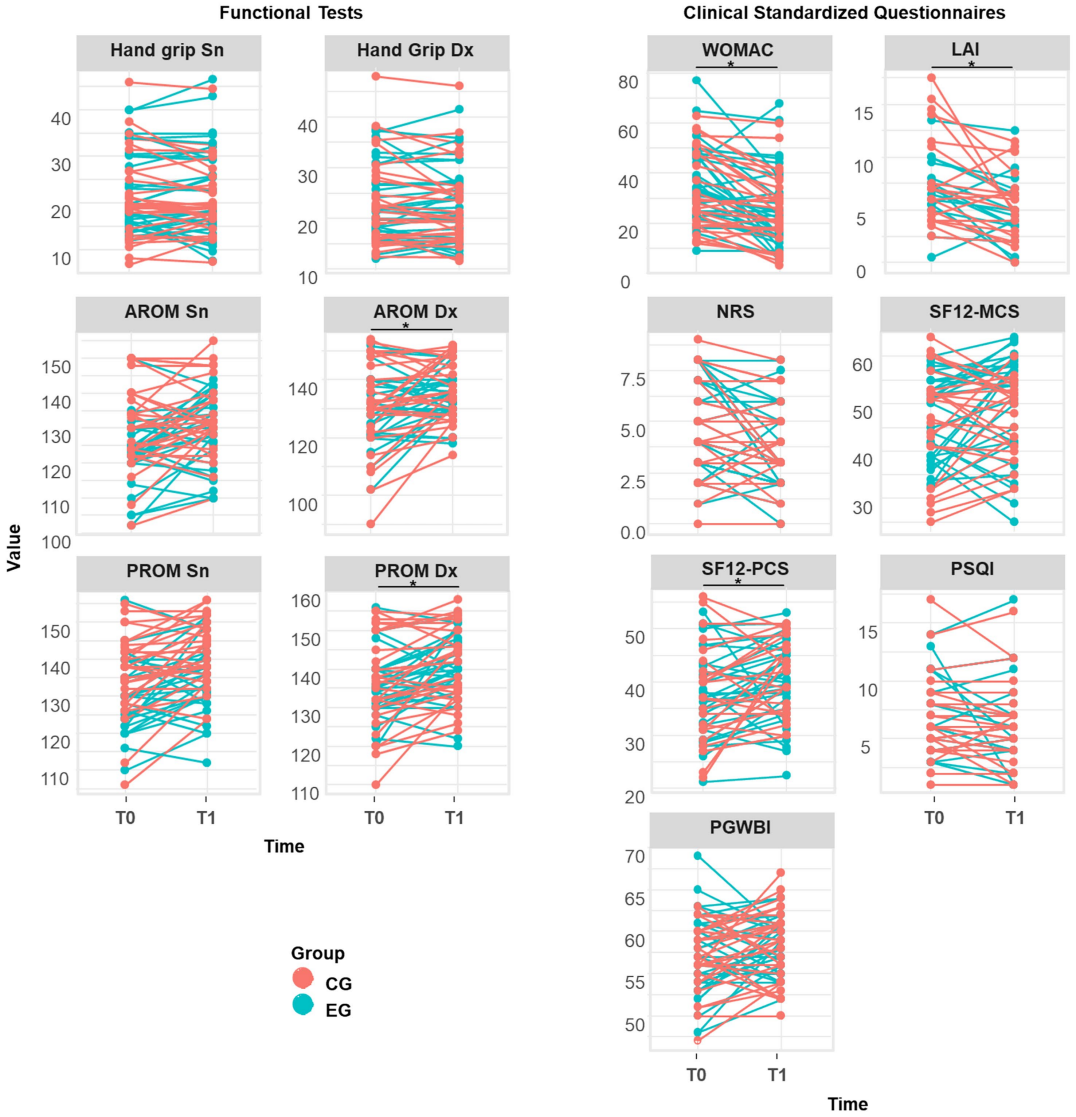
Change in the value of functional and patient-reported clinical outcome measures from T0 to T1 for each of control group (CG) in red and EG experimental group (EG) in blue. AROM: active range of motion. PROM: passive range of motion. NRS: Numerical Rating Scale. WOMAC: Western Ontario and McMaster Universities Osteoarthritis Index. LAI: Lequesne Algo-functional Index. SF12-PCS: Physical Component Summary of the SF-12 Health Survey. SF12-MCS: Mental Component Summary of the SF-12 Health Survey. PSQI: Pittsburgh Sleep Quality Index PGWBI.

When investigating the within-subject changes from baseline to the one-month follow-up, significant improvements were observed across several key functional parameters (Figure 4; Table 4). Both groups demonstrated a significant increase in passive range of motion for both the right and left sides. Specifically, the CG showed an increase of 3.6% on the right (*p* = 0.002) and 4.4% on the left (*p* = 0.001), while the EG reported improvements of 2.9% (*p* = 0.031) and 3.8% (*p* = 0.011), respectively. Regarding active range of motion, the EG exhibited significant gains on both sides, whereas the CG showed significant improvement only on the right side (*p* = 0.013). In contrast, no significant changes were recorded for hand grip strength in either group during the study period.

In terms of patient-reported outcomes, both groups showed significant improvements in pain intensity and functional capacity. The Numerical Rating Scale scores decreased in both groups, with a larger effect in the EG (r = 0.64) compared to CG (r = 0.42). Specifically, it decreased by 13% in the CG (*p* = 0.039) and by a more pronounced 31% in the EG (*p* = 0.001). Functional indices followed a similar positive trend, with WOMAC scores decreasing by 25% in the CG and 31% in the EG, both reaching statistical significance (*p* < 0.001). Similarly, the Lequesne Algo-functional Index scores were reduced by 31.5 and 29% for the CG (*p* = 0.009) and the EG (*p* = 0.017), respectively. Quality of life measures showed minor improvements, with the EG achieving a moderate gain in SF-12 mental health scores (r = 0.34, *p* = 0.063) and small-to-moderate changes in SF-12 physical scores, PSQI, and PGWBI. The SF-12 Physical Component Summary increased significantly in both the CG (+9.9%; *p* = 0.041) and the EG (+5.8%; *p* = 0.014). It is noteworthy that although the SF-12 Mental Component Summary did not show a significant time effect, the EG exhibited a relatively greater improvement of 8.2% compared to the 2% observed in the CG.

### 3.3 Linear mixed effects regression models

The linear mixed effects regression models indicated a significant effect of time for the active and passive range of motion of the left knee, confirming the baseline differences previously identified for the left knee’s mobility (Table 5). This indicates that active and passive ROM of the left knee were significantly different between EG and CG participants at baseline (at T0) (*p* = 0.003 for active ROM and *p* = 0.016 for passive ROM). When comparing the pre-treatment and post-treatment data, the model confirmed a significant time effect for the active ROM in the right knee (*p* = 0.008), the passive ROM in both knees (*p* < 0.003), indicating that these outcome variables significantly increased for CG participants post-treatment. A significant time effect for WOMAC (*p* < 0.001), LAI (*p* = 0.003), and SF-12 Physical Component Summary scores (*p* < 0.031) showed a significant decrease in WOMAC and LAI scores and a significant increase in SF12-PCS score after treatment for CG participants. No significant Time: Group interaction was found, suggesting that both intervention protocols followed a similar trend over the follow-up period for both groups. Therefore, ROM, WOMAN, LAI, and SF-12 scores improved significantly over time, and the change in these outcomes did not differ significanly between groups.

**Table 5:** Summary of the mixed effect regression models investigating changes in outcomes between time points (i.e., T0 vs. T1) while accounting for interindividual variability and participant characteristics.

DV	Intercept	age	BMI	Time	Group	Time:Group
Hand Grip Dx (kg)	23.57***	−2.14* (*p* = 0.036)	0.62	−1.10~ (*p* = 0.069)	−0.65	1.23
Hand Grip Sn (kg)	21.56***	−2.71* (*p* = 0.018)	0.92	−0.73	0.86	0.78
AROM Dx (°)	130.36***	−0.13	−5.99*** (*p* < 0.001)	5.83** (*p* = 0.008)	−0.95	−1.23
AROM Sn (°)	131.00***	−1.14	−4.72*** (*p* < 0.001)	2.24	−7.83** (*p* = 0.003)	3.76
PROM Dx (°)	136.88***	−0.40	−4.53*** (*p* < 0.001)	4.76** (*p* = 0.003)	−0.86	−1.13
PROM Sn (°)	136.26***	−0.44	−3.62** (*p* = 0.001)	5.52** (*p* = 0.001)	−5.98* (*p* = 0.016)	−0.90
NRS	4.52***	0.12	0.04	−0.69~ (*p* = 0.077)	0.72	−0.92~ (*p* = 0.084)
WOMAC	32.78***	−0.22	2.00	−8.17*** (*p* < 0.001)	4.12	−3.06
LAI	8.00***	0.12	0.52	−2.58** (*p* = 0.003)	−0.92	0.58
SF12-MCS	45.49***	−0.36	−0.47	0.96	0.96	2.92
SF12-PCS	37.69***	−0.37	−0.77	3.60* (*p* = 0.031)	0.02	−1.47
PSQI	6.50***	0.26	−0.21	−0.42	0.77	−0.40
PGWBI	56.81***	−0.14	0.51	1.45~ (*p* = 0.084)	0.42	−0.55

Intercept represents the expected value of the outcome measure for the CG at baseline (T0) for mean Age and BMI. The effect of *age* and *BMI* represent the association between *age* and *BMI* and the outcome variable: a significant *p*-value indicates a significant association. The main effect of *time* represents the change over time for the CG: a significant p-value indicates a significant change in the variable over times. The main effect of *Group* represents the difference between the EG and CG at baseline (T0): a significant p-value indicates a significant baseline difference between groups. The time*Group interaction tests whether the effect of *Time* differs between *Groups*: a significant *p*-value indicates that the change from T0 to T1 differs between the EG and CG. Asterisks indicate the level of statistical significance: *indicates *p* < 0.05, **indicates *p* < 0.01, ***indicates *p* < 0.001.

The regression analysis further clarified that age was negatively correlated with hand grip strength on both sides (*p* < 0.04), indicating that older participants tended to exhibit lower strength levels. Similarly, body mass index (BMI) showed a negative correlation with knee mobility, as higher values were significantly associated with lower active and passive ROM across both sides (*p* < 0.001).

## 4 Discussion

The primary objective of this study was to evaluate the clinical impact of a combined protocol integrating balneotherapy and AE compared to balneotherapy alone in patients with mild-to-moderate knee OA. Short-term improvements were recorded in both groups across several metrics, including NRS, WOMAC, LAI, and SF-12. However, the EG demonstrated a more substantial decrease in pain intensity and consistent improvements in bilateral AROM-.

The significant improvements observed in both groups regarding AROM and PROM, as well as WOMAC and LAI scores, confirm that balneotherapy is an effective option for conservative management of degenerative joint diseases (16, 25). The 25% decrease in the WOMAC score observed in the CG is consistent with results reported in similar protocols, such as those by Fioravanti et al. (26) and Varžaitytė et al. (27). Previous literature has established that balneotherapy reduces pain and stiffness while improving motor function through the anti-inflammatory, muscle-relaxant, and analgesic properties of mineral waters, with some effects lasting up to six months (28, 29). Studies showed that balneotherapy regulates immune responses by reducing pro-inflammatory mediators and matrix-degrading enzymes, while enhancing antioxidant defenses and chondroprotection, which helps preserve joint tissue and reduce pain (30, 31).

However, in our study the magnitude of the clinical response, particularly regarding pain reduction and functional scores, was significantly more pronounced in the combined treatment group (i.e., in EG), suggesting an additional effect due to the mechanical advantages of aquatic exercise. Water-based exercise interventions has repeatedly showed positive effects on patients with knee OA, with significant improvements reported in mobility, self-efficacy, and knee strength, as well in other health-related measures (32, 33). When compared to land-based exercise, aquatic programs generally provide similar improvements in pain and function but tend to have fewer adverse effects and better adherence (34).

In our study, the EG exhibited a 31% reduction in the NRS for pain, compared to a 13% reduction in the CG. AE have been shown effective in controlling pain across various chronic conditions by leveraging water’s unique properties such as buoyancy, hydrostatic pressure, and temperature, which reduce joint load and provide continuous sensory input that can modulate pain perception (35). Randomized controlled trials showed that AE significantly reduces pain intensity and improves quality of life in fibromyalgia, chronic low back pain, knee osteoarthritis, and Parkinson’s disease, often outperforming or matching land-based therapies with better patient adherence and fewer adverse effects (36, 37, 39). The sensory stimulation from water may activate neurophysiological mechanisms like the Gate Control Theory, where non-painful input inhibits nociceptive signals at the spinal level, creating a therapeutic window for active rehabilitation with less discomfort (40). Meta-analyses confirm AE reduces pain and improves physical function more effectively than no exercise and often better than land-based exercise, with benefits sustained up to 12 months in some conditions (35, 41).

Moreover, the integrated therapeutic approach yielded significantly more stable improvements in AROM. Although immersion in mineral waters functions as an effective “priming” phase by optimizing the viscoelasticity of periarticular structures - accounting for the equivalent gains in PROM observed in both groups - passive balneotherapy proved insufficient for restoring functional active excursion. The superior bilateral recovery of AROM within the EG could be explained by the superior efficacy of AE in addressing Arthrogenic Muscle Inhibition (42). Through the mechanism of joint unloading, water-based exercises attenuates the nociceptive reflexes that typically restrict motor unit recruitment, allowing for full-range active mobilization more difficult to achieve in traditional weight-bearing environments (43). Furthermore, the integrated protocol enhanced dynamic joint stability by leveraging hydrodynamic resistance and viscosity. Promoting quadriceps strengthening while refining proprioceptive input (44). Ultimately, AE could serve as a catalyst for functional rehabilitation, effectively bridging the divide between passive tissue extensibility and centralized neuromuscular integration.

While the SF-12, PGWBI, and PSQI did not reach formal statistical significance - likely due to the relatively short follow-up period and the outpatient basis of the treatments - a notable trend toward improvement was exclusively observed in the EG. Unlike passive balneotherapy, the active participation in a group setting encourages social support and peer modeling, which can enhance individual resilience and psychological well-being. Group-based interventions combining balneotherapy with psychoeducation have demonstrated reductions in caregiver burden and maladjustment, highlighting the added value of social interaction (45). Furthermore, the improvement trend in the EG suggests a potential reduction in kinesiophobia (fear of movement): by successfully performing movements in the buoyancy-assisted, pain-free environment of the thermal pool, patients experience a boost in self-efficacy, which is a key predictor of mental well-being in chronic musculoskeletal conditions (46). These results aligns with previous studies showing that aquatic physical activity improves mental health outcomes such as mood, fear of falling, and internal health locus of control in sedentary older adults (47). Compared to land-based exercise, AE offers a low-impact, supportive environment that may facilitate greater adherence and enjoyment, contributing to better mental health outcomes (48, 49).

Regression analysis provided further insight into the determinants of rehabilitation outcomes. The significant negative correlation between BMI and knee mobility confirms that elevated BMI constitutes a multifactorial barrier to functional recovery. Beyond the increased mechanical load on weight-bearing joints, obesity is characterized by a chronic, low-grade systemic inflammatory state (50, 51). This background inflammation likely attenuates the specific anti-inflammatory and antioxidant effects of sodium chloride–bromide–iodide thermal waters, leading to suboptimal therapeutic responses in obese subjects compared to normal-weight patients. Consequently, to maximize the efficacy of AE in this subgroup, it is recommended to implement a multidisciplinary approach that combines balneotherapy with targeted nutritional interventions aimed at reducing systemic inflammation (52). Similarly, the negative correlation between age and handgrip strength underscores the progressive impact of sarcopenia on functional decline, as showed by previous studies on aging population (53, 54). The analysis revealed a less pronounced therapeutic gain in the oldest age groups, suggesting that physiological responsiveness to rehabilitation stimuli diminishes with advancing age. This finding has important clinical implications, indicating that early referral to AE is crucial. Initiating treatment at a younger age, before sarcopenic changes become irreversible, allows patients to leverage a greater physiological reserve, thereby optimizing long-term rehabilitation outcomes.

Despite these positive outcomes, certain limitations must be acknowledged. The one-month follow-up period allows for the assessment of short-term efficacy but does not provide data on the long-term durability of these functional gains. Furthermore, the specialized setting of the Euganean Basin may limit the generalizability of these results to other clinical contexts.

In conclusion, this study demonstrates that a combined intervention in Health Resorts effectively reduces the clinical burden of knee OA in the short term. The integration of AE significantly enhances the analgesic and functional benefits of standard balneotherapy. These findings support the inclusion of combined thermal protocols in the comprehensive management of knee OA, providing a safe, non-pharmacological alternative that addresses both the biological and mechanical components of the disease.

## 5 Conclusion

In light of the rising prevalence of knee OA, the development of cost-effective and sustainable rehabilitation models is essential to address the escalating socioeconomic burden and associated patient disability (55). Specialized Health Resort settings offer a strategic, decentralized therapeutic model for managing chronic degenerative joint diseases, effectively reducing the clinical pressure on acute-care hospital infrastructures (38, 56). This study demonstrates that an intensive integrated treatment - combining the biochemical benefits of balneotherapy with the physical-mechanical advantages of AT - provides a superior therapeutic outcome for knee OA in terms of pain and functional mobility. The identification of elevated BMI and advanced age as limiting factors suggests that efficacy could be significantly enhanced by integrating nutritional strategies to counteract systemic inflammation in obese patients and prioritizing early-stage OA interventions to exploit the greater physiological reserves of younger subjects. These findings advocate for the inclusion of combined Health Resort interventions in personalized, non-pharmacological management strategies aimed at preserving joint longevity and optimizing patient quality of life. Additional studies are needed to investigate the long-term effects of the combined protocol compared to balneotherapy alone.

## Data Availability

The original contributions presented in the study are included in the article/supplementary material, further inquiries can be directed to the corresponding author.
